# Heat stress and strain limits applicable to military helicopter aircrew

**DOI:** 10.1186/2046-7648-4-S1-A26

**Published:** 2015-09-14

**Authors:** Andy Weller, Jonathan Boyd, Ken Puxley

**Affiliations:** 1 Aircrew Systems, QinetiQ, Cody Technology Park, Ively Road, Farnborough, GU14 0LX, UK

## Introduction

Heat stress can result in uncompensated heat storage (termed heat strain and indicated by core temperature, *T_C_*), heat illness, impaired performance and fatigue, and represents a significant threat to the effectiveness of aircrew undertaking hot-weather operations. To determine the potential risk to aircrew, two physiological aircrew simulation trials (1 & 2) were undertaken to assess the environmental heat stress resulting in a mean *T_C _*of 38 °C (occupational limit [*e.g*. 1]), and the level of parallel heat strain variables.

## Methods

In Trial 1, 16 unacclimatised men undertook 4 × 170-minute heat tests in a thermal chamber controlled at wet-bulb-globe-temperatures (WBGTs) of 18, 22, 27 and 31 °C wearing a representative aircrew clothing and equipment ensemble (including body armour). The volunteers were assigned to two groups of 8, with one group completing an exercise protocol representative of the metabolic cost of helicopter pilots (Front) and the other undertook a rear crew simulation (Rear) (metabolic rate 95 and 117 W.m^-2^, respectively). In Trial 2, 8 different men completed the heat test at a WBGT of 28.5 °C. Physiological (rectal temperature, *T_RE_*; mean skin temperature, *T_SK_*; total rate of mass loss, *M_TOT_*; heart rate, *HR*), and subjective indicators (thermal sensation, *TS*; thermal comfort, *TC*) of heat strain were obtained in both trials.

## Results

Mean heat strain responses at 170 minutes in the different thermal environments for Front are given in Table 1. The responses were generally not different between Front and Rear.

**Table 1:** Mean heat strain responses in the 5 thermal environments

Variable / WBGT (°C)	18	22	27	28.5	31
*T_RE _*(°C)	37.5	37.4^ns^	37.7**^##^**	38.1**^§^**	38.5**^¥^**

*T_SK _*(°C)	34.1	35.2**^ΔΔ^**	36.4**^##^**	37.4**^§§^**	37.3^ns^

*M_TOT _*(kg.h^-1^)	0.2	0.3**^Δ^**	0.4**^#^**	0.6**^§§^**	0.7^ns^

*HR*/↑*HR *[vs WBGT 18] (b.min^-1^)	88/0	91^ns^/3^ns^	103/14**^##^**	118/30**^§^**	129/41^ns (p = 0.08)^

*TS*^1^/*TC*^2^	1.1/0.5	1.4^ns^/0.8^ns^	2.1**^#^**/0.9^ns^	3.0**^§^**/2.1**^§§^**	3.3^ns^/2.1^ns^

^1^Thermal Sensation (*TS*) ratings: 0 (Neutral), 1 (Slightly Warm), 2 (Warm), 3 (Hot), 4 (Very Hot). ^2 ^Thermal Comfort (*TC*) ratings: 0 (Comfortable), 1 (Slightly Uncomfortable), 2 (Uncomfortable), 3 (Very Uncomfortable), 4 (Exceedingly Uncomfortable). Statistical differences between WBGT 18 and 22 (^Δ^), 22 and 27 (^#^), 27 and 28.5 (^§^) and 28.5 and 31 (^¥^) are given by single (P < 0.05) and double (P < 0.01) symbols (Students t tests). ^ns ^signifies no statistical difference.

## Discussion

There was a general curvilinear relationship between WBGT and the heat strain responses. The 38 °C *T_C _*limit was reached at 165 minutes in the 28.5 °C environment and the corresponding increase in *HR *was consistent with the physiological limit in ISO 9886 [1]. It remains to be established whether the subjective thermal strain experienced at a WBGT of 28.5 °C (partly driven by high T_SK_) could influence performance.

## Conclusion

Helicopter aircrew are likely to exceed the *T_C _*limit of 38 °C and be at risk of heat-related illness and impaired performance when exposed to a cockpit/cabin WBGT of ~28 °C and beyond.
